# In Situ Reflectometry and Diffraction Investigation of the Multiscale Structure of p-Type Polysilicon Passivating Contacts for c-Si Solar Cells

**DOI:** 10.1021/acsami.2c01225

**Published:** 2022-03-31

**Authors:** Audrey Morisset, Theodosios Famprikis, Franz-Josef Haug, Andrea Ingenito, Christophe Ballif, Lars J. Bannenberg

**Affiliations:** † Photovoltaics and Thin Film Electronics Laboratory, Institute of Electrical and Microengineering (IEM), Ecole Polytechnique Fédérale de Lausanne (EPFL), Maladière 71b, 2002 Neuchâtel, Switzerland; ‡ Department of Radiation Science and Technology, Faculty of Applied Sciences, Delft University of Technology, Mekelweg 15, 2629JB Delft, The Netherlands; § Sustainable Energy Center, CSEM, Rue Jaquet-Droz 1, Neuchâtel, 2002, Switzerland

**Keywords:** c-Si solar cells, passivating contacts, poly-Si, SiO_*x*_, X-ray reflectometry, in situ monitoring, annealing

## Abstract

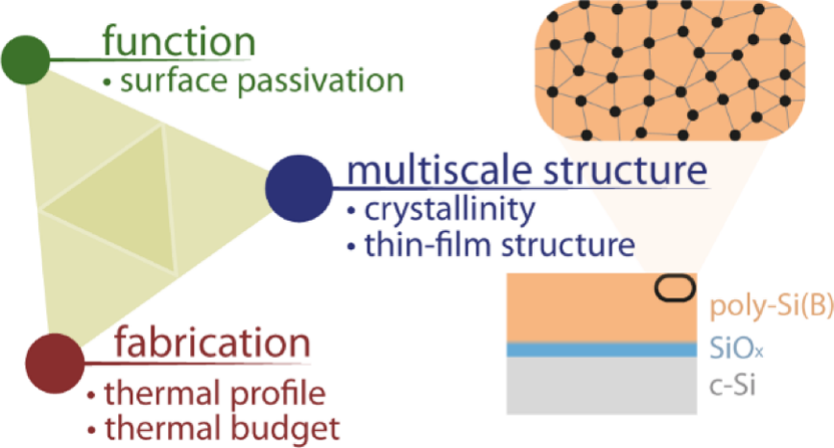

The integration of passivating contacts based on a highly doped polycrystalline silicon (poly-Si) layer on top of a thin silicon oxide (SiO_*x*_) layer has been identified as the next step to further increase the conversion efficiency of current mainstream crystalline silicon (c-Si) solar cells. However, the interrelation between the final properties of poly-Si/SiO_*x*_ contacts and their fabrication process has not yet been fully unraveled, which is mostly due to the challenge of characterizing thin-film stacks with features in the nanometric range. Here, we apply in situ X-ray reflectometry and diffraction to investigate the multiscale (1 Å–100 nm) structural evolution of poly-Si contacts during annealing up to 900 °C. This allows us to quantify the densification and thinning of the poly-Si layer during annealing as well as to monitor the disruption of the thin SiO_*x*_ layer at high temperature >800 °C. Moreover, results obtained on a broader range of thermal profiles, including firing with dwell times of a few seconds, emphasize the impact of high thermal budgets on poly-Si contacts’ final properties and thus the importance of ensuring a good control of such high-temperature processes when fabricating c-Si solar cells integrating such passivating contacts. Overall, this study demonstrates the robustness of combining different X-ray elastic scattering techniques (here XRR and GIXRD), which present the unique advantage of being rapid, nondestructive, and applicable on a large sample area, to unravel the multiscale structural evolution of poly-Si contacts in situ during high-temperature processes.

## Introduction

Photovoltaic (PV) technologies are among the key sources of energy to support the transition toward a 100% renewable energy scenario to reduce human-related carbon emission and mitigate global warming. PV technologies based on crystalline silicon (c-Si), which currently represent ∼95% of the global market, will be the main driving force toward the expected growth of worldwide PV installations up to the TW scale.^1,2^ One straightforward way to support this transition is to improve c-Si solar cell conversion efficiencies while avoiding disruptive changes to tools and processes currently implemented in mainstream production lines. Lately, the further increase of c-Si solar cell efficiency has mostly relied on the integration of “passivating contacts”, which consists in stacks of thin films introduced at the metal/c-Si interface to decrease efficiency losses related to these defect-rich interfaces.^3,4^ One of the most promising passivating contacts to rapidly bridge the gap between device efficiencies in R&D and those in production is based on a highly doped polycrystalline silicon (poly-Si) layer on top of a thin silicon oxide (SiO_*x*_) buffer layer.^5^ Due to its resilience at high temperatures, the poly-Si/SiO_*x*_ contact promises compatibility with the mainstream metallization process currently used in the industry and thus a rapid increase of the c-Si solar cell efficiency beyond 24% in mass production.^5−7^

The key steps to fabricate such a poly-Si contact are the following: first, a thin SiO_*x*_ layer (∼1–2 nm) is grown at the c-Si surface, followed by the deposition of a silicon (Si) layer (either amorphous or already polycrystalline) that may be alloyed with additional elements, for example, carbon or oxygen.^8−10^ Doping of the Si layer, most commonly with boron or phosphorus, can be performed either in situ during layer deposition or through a subsequent step to obtain a hole- or an electron-selective contact, respectively.^11−14^ Then, an annealing at high temperature (800–1100 °C) is performed to further crystallize the Si layer and to activate its doping. Finally, normally combined with the metallization, the passivating contact is hydrogenated by a so-called “hydrogenation” step which commonly consists in the deposition of a hydrogen-rich dielectric layer [e.g., silicon nitride (SiN_*y*_/H)] and subsequent rapid annealing referred to as “firing” to diffuse hydrogen through the structure.^15,16^ The interplay between different mechanisms during these key process steps (especially high-temperature annealing and subsequent hydrogenation) along with the challenge of characterizing thin-film stacks and buried layers and interfaces has limited the understanding of poly-Si contacts’ working principle, resulting in optimization mostly based on “trial-and-error”. Novel characterization methodologies and in situ monitoring could provide better insights into the interrelation between poly-Si contacts’ properties and their fabrication process, ultimately driving the fabrication of better passivating poly-Si contacts and thus solar cells with higher efficiency.

A prominent example of a key process step whose effect on the poly-Si final properties is not completely understood yet is the high-temperature annealing performed to “activate” the formation of the poly-Si contact. Other than crystallization and doping activation, such high-temperature annealing has been shown to cause a shallow diffusion of dopants into the underlying c-Si substrate (and thus through the thin SiO_*x*_ layer) as well as chemical/structural changes of the thin SiO_*x*_.^17−19^ Moreover, lately, several studies have emphasized the detrimental impact of firing on the surface passivation properties provided by poly-Si contacts.^20−22^ It is worth noting that in the currently foreseen integration of poly-Si contacts in c-Si solar cells, it is most likely that poly-Si contacts will be submitted to a firing process applied at the very end of the solar cell fabrication to contact the metal paste to the c-Si and/or poly-Si layer.^6,23^ Thus, it is of utmost importance to better understand the impact of such high-temperature processes on the final properties of poly-Si contacts.

Here, we applied X-ray reflectometry (XRR), a nondestructive technique probing thicknesses and densities of thin films in multilayer structures, and grazing-incidence X-ray diffraction (GIXRD) to investigate the multiscale (0.1–100 nm) structure of poly-Si contacts. More particularly, in situ characterization was performed to monitor the structural evolution of both the poly-Si layer and the buried SiO_*x*_ interface during annealing up to 900 °C. In the following, we first elaborate on the experimental methods used. Then, we describe the results obtained by in situ XRR and GIXRD measurements of boron-doped poly-Si/SiO_*x*_ contacts. Finally, we further discuss the structural evolution of our poly-Si contact during high-temperature annealing and we investigate the impact of a broader range of thermal profiles on the poly-Si contact structural properties.

## Experimental Methods

### Sample Preparation

For the purpose of this study, symmetrical samples featuring passivating contacts on both sides of c-Si substrates were fabricated for characterization notably by means of XRR and XRD. To this end, samples featuring the flattest surfaces possible are required; thus, we used phosphorus-doped (100)-oriented 4 inch FZ c-Si wafers with mirror-polished surfaces (i.e., roughness < 1 nm), with a thickness of 280 μm and a resistivity in the range 1–5 Ω cm. Additional samples were fabricated from phosphorus-doped (100)-oriented 4 inch FZ c-Si wafers with shiny-etched surfaces (i.e., roughness of about 15 nm), featuring a thickness of 200 μm and a resistivity of about 2 Ωcm to evaluate the surface passivation properties by means of photo-conductance decay (PCD) measurements.

**Figure 1:**
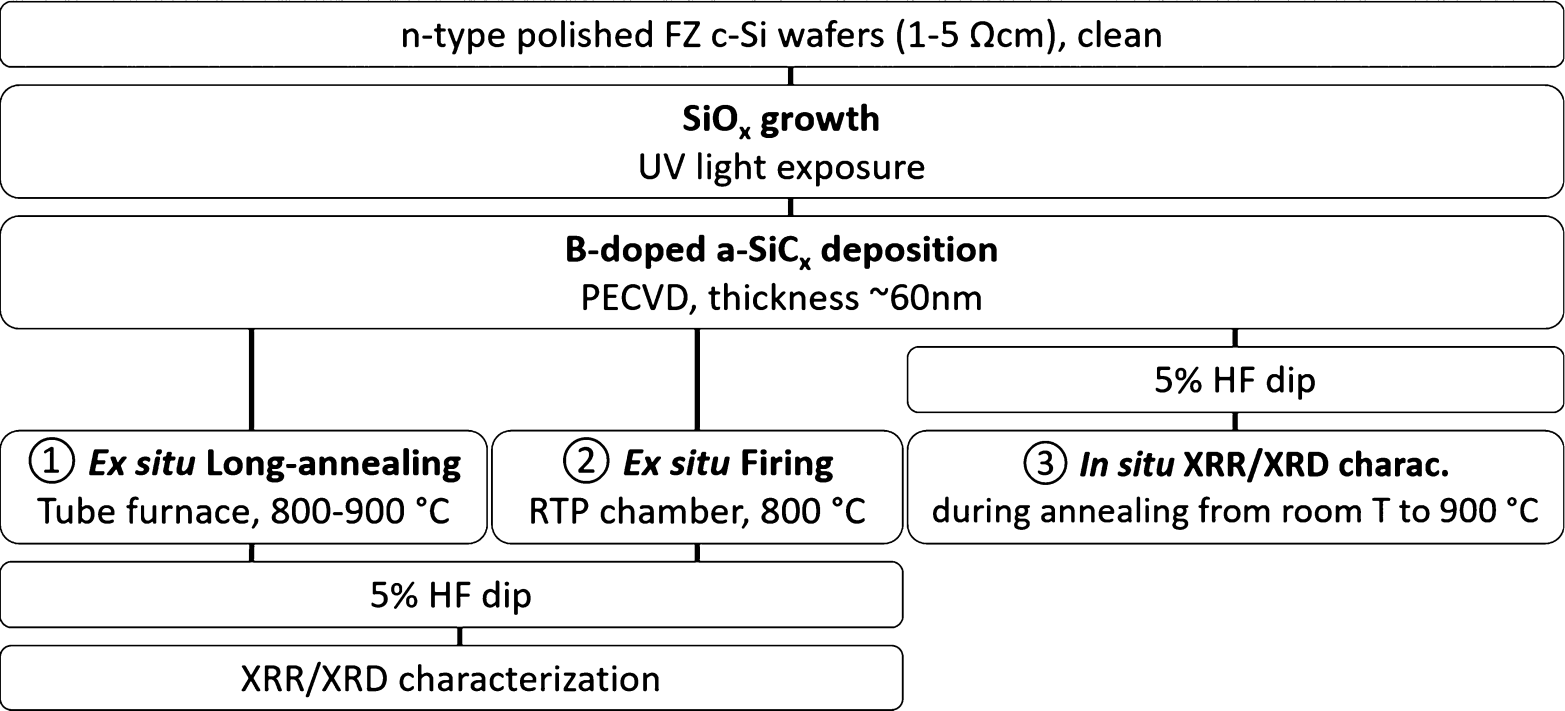
Flowchart of samples’ preparation and characterization.

The main steps for samples’ preparation and characterization are summarized in Figure 1. The sample preparation was initiated by a standard RCA cleaning of the c-Si wafers.^24^ A thin SiO_*x*_ layer was then grown on both sides of the wafers by first dipping them in a 5% HF solution for 1 min, followed by exposure to UV light for 2 min on each side (Jelight, UVO cleaner42). A thickness of about 1 ± 0.3 nm was evaluated by spectroscopic ellipsometry for the resulting SiO_*x*_ layer right after growth. Hydrogenated boron-doped silicon layers containing approximately 3–5 at. % of carbon [denoted “a-SiC_*x*_(p)”] were then deposited on both sides by PECVD at a temperature of 200 °C using silane, methane, hydrogen, and trimethylboron (TMB) as precursor gases. A CH_4_-normalized flow ratio [*r* = CH_4_/(H_2_ + CH_4_ + SiH_4_ + TMB)] of 0.1 was applied for deposition of the a-SiC_*x*_ layer. In the following, this first type of sample right after deposition of a-SiC_*x*_(p) layers on both sides will be referred to as “as-deposited” samples. Subsequently, two annealing methods were employed to form the poly-Si contact, that is, crystallize the a-SiC_*x*_ layer (then denoted “poly-Si” layer) and activate the boron dopants incorporated in the layer during deposition. A first set of samples was annealed in a quartz tube furnace (PEO-603, ATV) in the range 800–900 °C under an argon atmosphere to form the poly-Si contact. For this “long-annealing” process, a heating ramp rate of 10 °C/min was applied to reach the targeted temperature, directly followed by a cooling ramp rate of 2 °C/min, that is, without holding a dwell time at the targeted temperature. A second set of samples was annealed in a rapid-thermal process (RTP) chamber featuring an IR lamp (JetFirst 200, Jipelec) at 800 °C under a nitrogen atmosphere. In this case, a “firing” thermal profile was targeted, and thus, a rapid heating ramp rate of 50 °C/s was applied up to 800 °C, followed by a dwell time in the range 2–200 s before cooling, resulting in an overall annealing time of a few minutes. Following high-temperature annealing, samples based on shiny-etched c-Si substrates were submitted to a so-called “hydrogenation process” consisting of the deposition of a hydrogen-rich silicon nitride (SiN_*y*_/H) layer by PECVD, followed by a firing step performed in a belt furnace (CAMiNI, Roth and Rau) with a target peak temperature of approximately 840 °C.

Before further characterization, the samples were dipped in a 5% HF solution for 1 min to remove the native oxide layer growing at their surface during exposure to ambient air [e.g., after a-SiC_*x*_(p) deposition and/or annealing]. Samples were then immediately stored in nitrogen- or argon-filled gloveboxes or transfer modules to limit the regrowth of any native oxide at their surface. Overall, air exposure was limited to <5 min in between HF cleaning and transfer to the chamber of the XRD setup and start of the measurement. Alternatively, a third set of samples was HF-dipped right after the deposition of a-SiC_*x*_ layers, loaded into the transfer module, and mounted to the in situ heating module of the XRD setup. In this last configuration, the sample was placed on a ceramic holder and heated through resistive elements located on the inner wall of the Anton Paar XRK 900 dome.

A flowchart of the different processes and characterization steps is shown in Figure 1, and the key characteristics of the different annealing setups used are summarized in Table 1.

**Table 1:** Key Characteristics of Different Annealing Setups Utilized in This Work

process	equipment	atmosphere	heating method	ramp rate	dwell time	sample holder
long annealing	Quartz tube furnace	Ar	resistive elements	10°C/min	none	quartz rack
	(PEO, ATV)		(convection + radiation)			
firing	RTP processor	N_2_	IR lamp	3000 °C/min	2–200 s	quartz pins
	(JetFirst 200, Jipelec)		(radiation)			
in situ annealing	reactor chamber	vacuum	resistive elements	12 °C/min	1–2 h	ceramic holder
	(XRK 900, Anton Paar)		(radiation)			

### Reflectometry

The XRR experiments were performed with a Bruker D8 Discover (Cu-Kα, λ = 0.154 nm) equipped with a LYNXEYE XE detector, in combination with a Goebel mirror, a 0.1 mm slit on the primary side, and two 0.1 mm slits on the secondary side. Via preliminary measurements in air, it was determined that the reflectograms were sensitive to the native oxide growing at the samples’ surface upon exposure to ambient conditions (see Figure S1 in the Supporting Information). Thus, all the reflectograms presented in the main text (including ex situ) were measured on HF-cleaned samples under vacuum (*p* < 5 × 10^–4^ mbar) in an Anton Paar XRK900 Reactor chamber. In situ XRR measurements were performed during annealing of “as-deposited” samples up to 900 °C. For in situ annealing, a heating ramp of 12 °C/min was applied from room temperature, followed by a 30 min dwell time at each temperature of interest up to 900 °C (10 min stabilization + 20 min measurement).

Measurements were performed with a 0.1 mm Cu absorber for 0 < 2θ < 2° and without an absorber for 0.6 < 2θ < 6°. Subsequently, the measurements were stitched together using a home-written Python code. The reflectograms were fitted with GenX3 (ref (25)) using a model including two layers on top of a c-Si substrate, namely, a top Si layer (a-Si or poly-Si depending on processing stage) and thin SiO_*x*_ layer at the interface. The substrate roughness and electronic density were fixed at values of 0.3 nm and 0.717*r*_e_/Å^3^, respectively, determined by measurements of bare substrates. The thickness, density, and roughness of the SiO_*x*_ and a-Si/poly-Si layers were extracted from the fits. All error bars reported correspond to the 68% confidence interval (1 standard deviation) and have been obtained from GenX.

### Grazing-Incidence X-ray Diffraction

The GIXRD measurements were performed on the same samples and instrument (Bruker D8 Discover; Cu-Kα, λ = 0.154 nm) using a primary 0.1 mm slit in combination with a 2.5° Soller slit on the detector side. The in situ measurements while heating were performed inside an Anton Paar XRK900 Reactor chamber under vacuum (*p* < 5 × 10^–4^ mbar) with an incidence angle of 0.2°. The incidence angle was optimized to minimize scattering contributions from the substrate (see Figure S2 in the Supporting Information).

### Photo-Conductance Decay

PCD measurements were performed on symmetrical samples made from shiny-etched c-Si substrates using a WCT-120 tool from Sinton Instruments to assess the implied open circuit voltage (iV_oc_) after long annealing at different *T* values (800–900 °C). In addition to iV_oc_, the emitter recombination current density (*J*_0_) associated to a single surface was also extracted according to the method of Kane and Swanson.^26^

## Results

### Comparison of As-Deposited and Annealed Samples

In this first part, we present and compare the XRR and GIXRD results obtained at the initial and final steps of the high-temperature annealing process, namely, after deposition of the a-SiC_*x*_ layer (“as-deposited”), after ex situ long annealing at 850 °C, and after in situ annealing up to 800 °C in the XRR/XRD chamber.

### Basics of Reflectometry

Reflectometry relies on analyzing the intensity of reflected radiation (here, X-rays) off of a flat surface as a function of angle, θ, (reflectogram) to elucidate the structure of thin films with layers typically in the range of about 1–200 nm. Often, the (magnitude of the) wavelength-independent scattering vector, *Q*, is used instead of reflection angle in the abscissa to allow comparison of data collected using radiation with different wavelengths, λ [*Q* = 4π sin(θ)/λ; units of inverse length]. In a multilayer structure, such as the object of this study, X-rays can reflect off of the multiple interfaces between layers and their regular spacing causes a regular pattern of constructive and destructive interference as a function of *Q*. The latter interference results in characteristic periodic fringes in the reflectogram, whose period is inversely related to the thickness and amplitude related to the density difference between the layers. Because X-rays interact with electrons in matter, in the case of XRR, the amplitude of the fringes relies on the difference in electronic density between subsequent layers. Furthermore, interfacial or surface roughness can cause dampening of the fringes, which can also be determined and attributed to the different layers based on the Q-dependence of said dampening. Fits of reflectograms can be visualized as a scattering length density (SLD) profile, typically plotted as a function of the distance from the substrate. In the case of X-rays, the SLD is equal to the electronic density (∼mass density), so changes in SLD can directly indicate a change of phase in a multilayer.^27,28^

**Figure 2:**
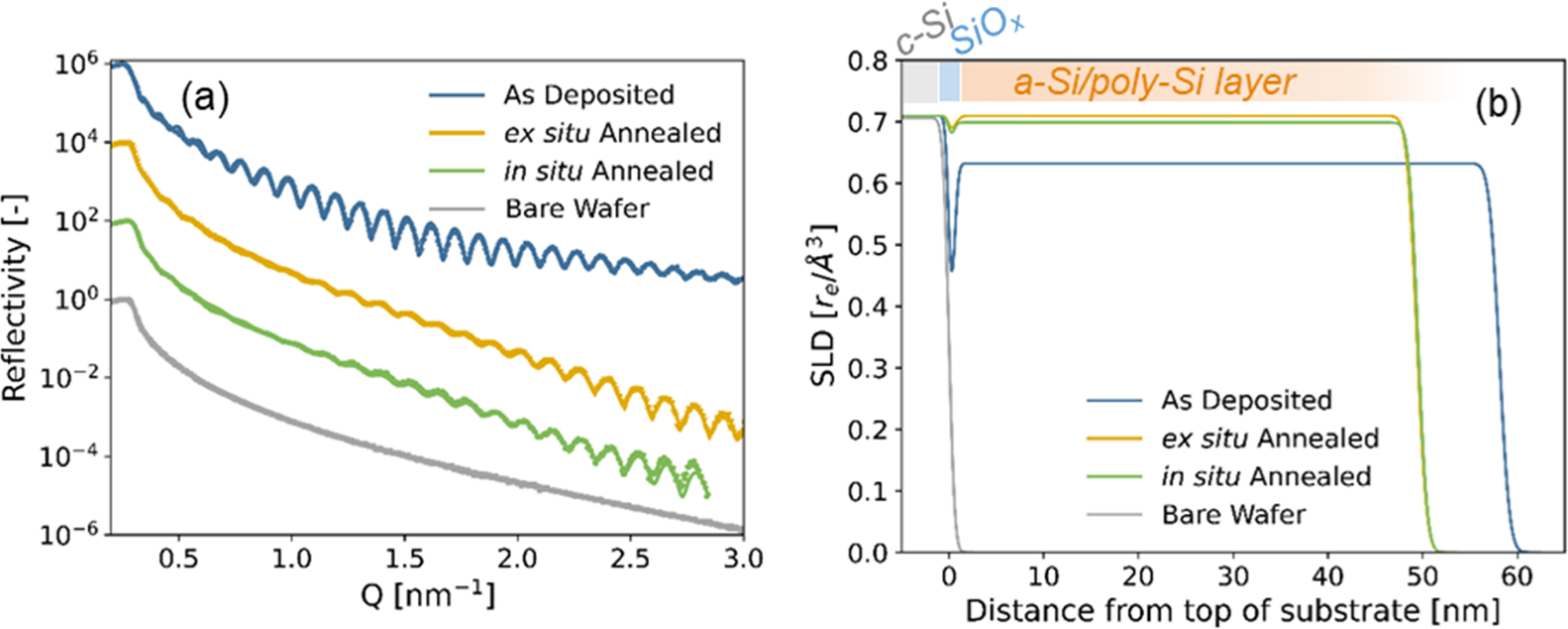
(a) X-ray reflectograms of samples at different processing stages and (b) associated SLD profiles in direct space [reconstructed from the fit of the reflectograms plotted in (a)]. ‘Ex situ’: long-annealed at 850 °C in tube furnace under Ar; ‘in situ’: annealed at 800 °C in X-ray chamber under vacuum. Structural parameters corresponding to the fits are tabulated in Table S1. Reflectograms in (a) are vertically shifted by a constant factor (×100) for clarity.

Figure 2a compares the reflectograms of the as-deposited, ex situ- and in situ annealed samples. The characteristic periodic fringes are well fitted using a two-layer model of a top Si layer (a-Si or poly-Si) and a thin SiO_*x*_ layer at the interface with the substrate. The resulting SLD profiles, plotted as a function of distance from the substrate, are visualized in Figure 2b. Significant changes are observed between as-deposited and annealed samples. First, the period between the fringes increases, indicating a reduced thickness (by about 10 nm) of the poly-Si layer after annealing. Second, the amplitude of the oscillations decreases along with the overall reflected intensity at *Q* > 2 nm^–1^, indicating the loss of contrast due to the densification of the SiO_*x*_ and poly-Si layers that both approach the density of the c-Si substrate.

The ex situ and in situ annealed samples appear virtually identical from the point of view of XRR, validating the approach of studying the effect of annealing in situ in the following part. We note that we observed a slight shift of ∼50 °C in the nominal temperature at which the different phenomena occur in the different setups. Namely, the ex situ annealed sample at 850 °C best resembles in structure the in situ annealed sample at 800 °C. This nominal offset will be discussed later on in the context of different heating apparatus and temperature profiles.

### Basics of Bragg Diffraction

Bragg X-ray diffraction (XRD) operates on a similar principle to reflectometry but at a different scale. Whereas in reflectometry, X-rays are reflected at low angles by differences between layers of material that can be as thick as >100 nm, in diffraction the relevant surfaces causing “reflections” are atomic planes in the scale of Å. Peak positions are characteristic of interatomic distances and define the crystal structure, while peak widths contain information on microstructural features such as crystallite size and microstrain.^29^ In grazing incidence geometry (GIXRD), it is possible to limit the penetration depth of the X-rays so as to study a thin film on top of a substrate by fixing the X-ray source at a small (“grazing”) incidence angle—here 0.2°—and rotate only the detector during measurements.

**Figure 3:**
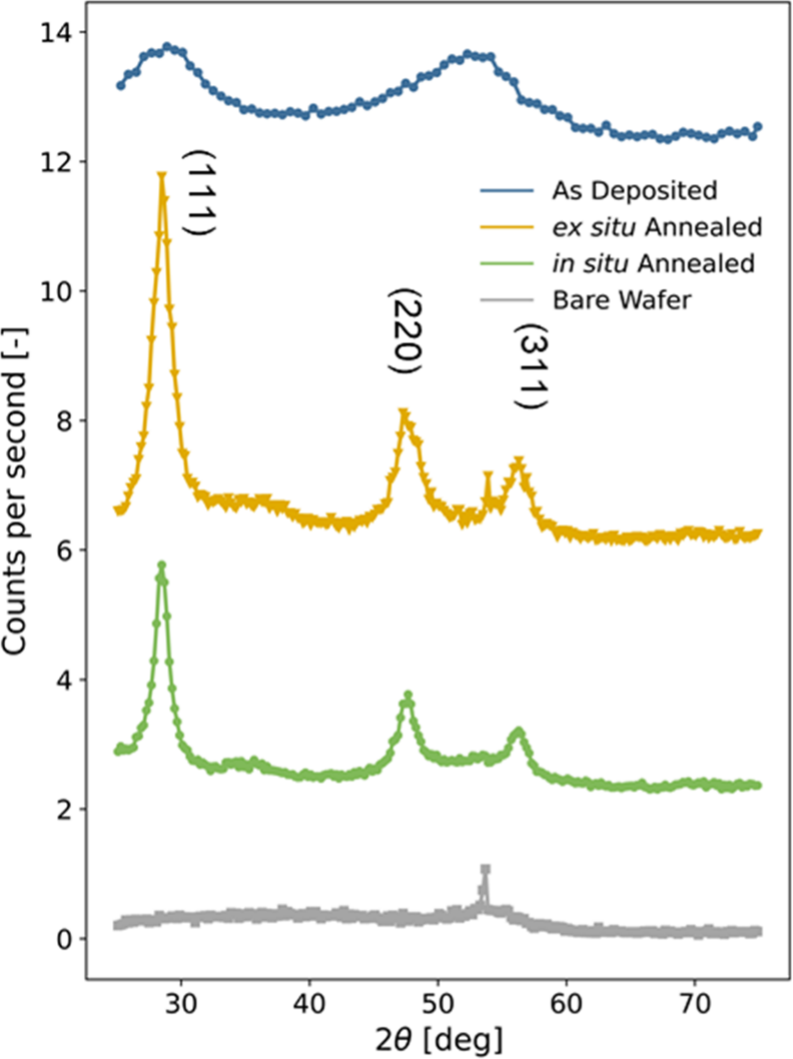
X-ray diffractograms of samples at different processing stages. Diffractograms are vertically shifted for clarity. The sharp peak at 2θ ≈ 53° visible in some patterns likely originates from an imperfection of the substrate and is unrelated to the layers of interest.

Figure 3 compares the diffractograms of the as-deposited, as well as the ex situ and in situ annealed samples measured on the same samples whose reflectograms are presented in Figure 2. The diffractograms obtained from the as-deposited samples feature only diffuse features centered around ∼28 and 52° that cannot be unambiguously assigned to Bragg positions of Si, confirming that the a-SiC_*x*_ layer is deposited in an amorphous state. Upon annealing, the overall diffracted intensity increases dramatically, indicating the precipitation of crystalline domains in the Si layer, which can be described as polycrystalline (poly-Si). The in situ and ex situ annealed samples show similar characteristics, justifying again our experimental procedure for in situ tracking of the effects of annealing on the structure. In both cases, three peaks of Si can be clearly observed at 2θ = 28.4, 47.3, and 56.1°; which can be attributed to Bragg diffraction from the (111), (220), and (311) planes of the silicon crystal structure, respectively (space group *Fd*3̅*m*).^30^ An additional sharp unindexed peak at 2θ ∼ 53° does not originate from the sample, as it is already present in the measurement of the bare substrate, and is attributed to an artifact of the measurement setup (see Figure S3 in the Supporting Information). We note that the bare substrate (⟨100⟩-oriented c-Si wafer) also exhibits a certain intensity for the (311) peak (see Figure S3 in the Supporting Information), so that the latter is the additive contribution of diffraction from the substrate and the poly-Si layer.

### In situ Characterization during Annealing of the Poly-Si/SiO_*x*_ Contact

**Figure 4:**
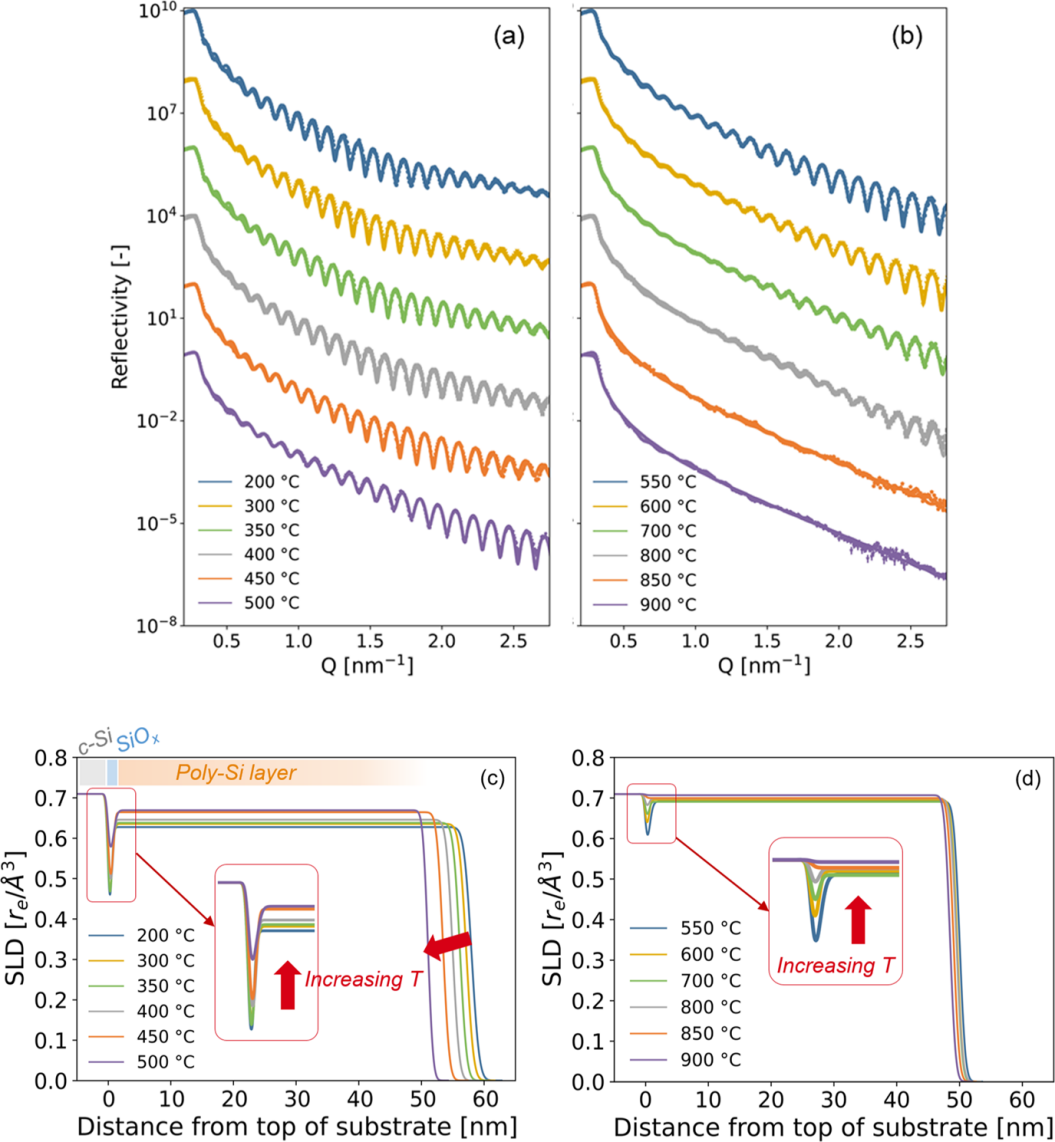
(a,b) X-ray reflectograms measured during in situ annealing of the as-deposited sample from room temperature to 900 °C and (c,d) associated SLD profiles obtained from the fits of the reflectograms. Structural parameters corresponding to the fits are tabulated in Table S2. Reflectograms in (a) and (b) are vertically shifted by a constant factor (×100) for clarity.

To further investigate the structural changes of the samples during annealing, in situ XRR and GIXRD measurements were performed during the annealing of as-deposited samples under vacuum. XRR reflectograms were recorded between 200 °C and 900 °C, and the resulting reflectograms and SLD profiles are illustrated in Figure 4.

**Figure 5:**
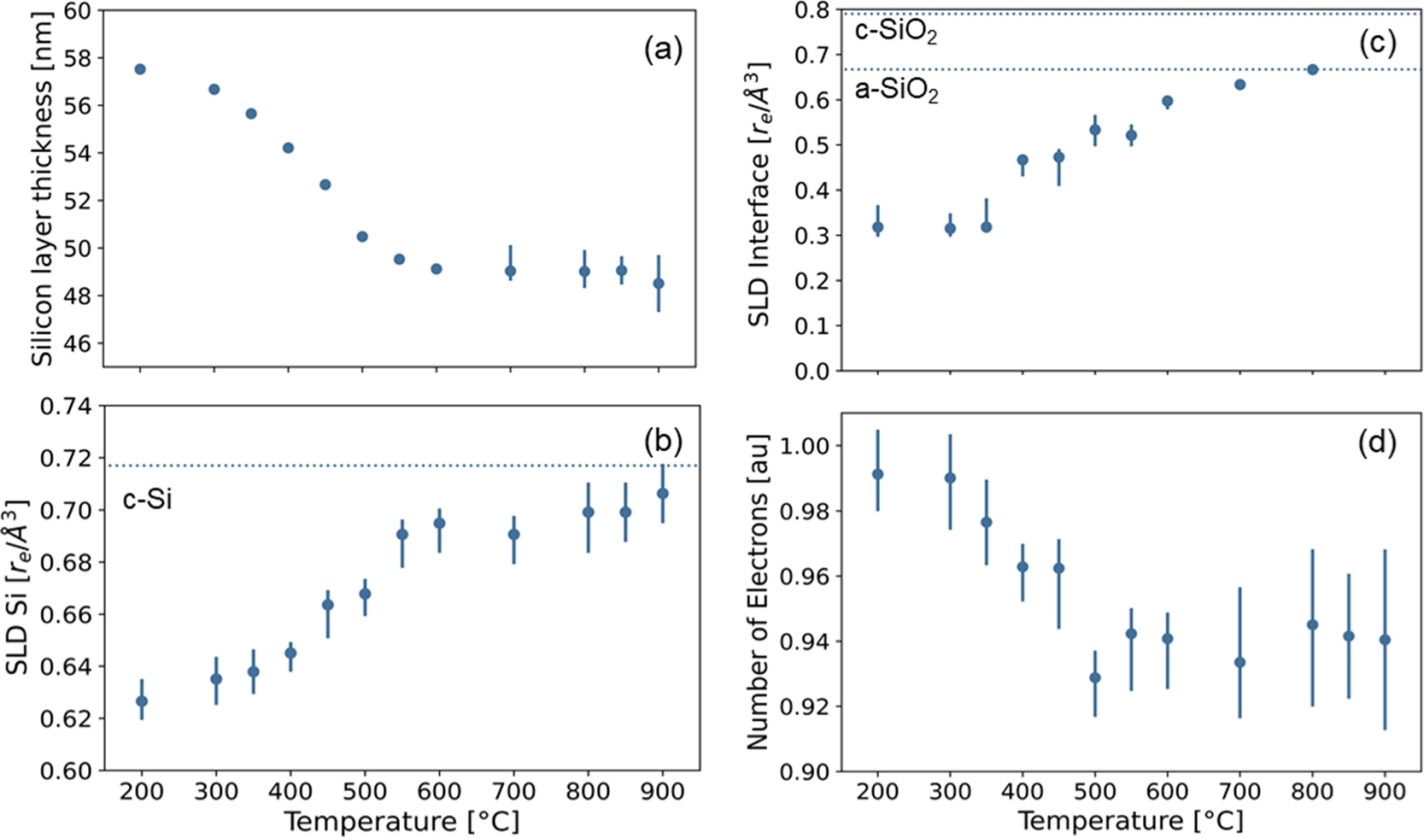
Parameters obtained from fitting the in situ reflectograms as functions of temperature: (a) thickness and (b) SLD associated to the poly-Si layer, (c) SLD of the SiO_*x*_ interfacial layer, and (d) summation of total electrons in the sample. Representative reference values for crystalline and amorphous quartz in panel (c) assume SiO_2_ stoichiometry and 2.65 and 2.20 g/cm^3^, respectively. We note that for some values, the error bars are smaller than the marker.

For temperatures from 200 °C to 400 °C, only few changes were observed on the X-ray reflectograms in terms of fringe period and amplitude. However, for *T* > 400 °C, the period between the fringes starts visibly increasing with the increase in temperature along with the decrease in amplitude of the oscillations and decreasing reflected intensity. For *T* > 800 °C, the amplitude of the oscillations almost completely vanished. As previously mentioned, these observations indicate both the reduction of the poly-Si layer thickness as well as the loss of contrast due to the densification of the SiO_*x*_ and poly-Si layers, approaching the density of the substrate. The associated SLD profiles obtained from fitting the reflectograms with the two-layer model are depicted in Figure 4c,d. The fitted parameters (thickness and density) associated to both poly-Si and SiO_*x*_ layers are given in Table S2 in the Supporting Information and some selected parameters are represented as a function of temperature in Figure 5. This latter representation enables us to observe that both layers became thinner and denser with the increase in temperature during in situ annealing.

For the poly-Si layer, these changes are most pronounced for temperatures from 200 °C to 500 °C with thickness decreasing from ∼60 to ∼50 nm and density almost reaching the one of the c-Si substrate. For *T* > 500 °C, the poly-Si layer thickness and density are then staying constant with the increase in temperature. For the SiO_*x*_ layer, one can observe a steady decrease of thickness and a simultaneous increase in density (Figure 5c) throughout the investigated temperature range. Due to the strong correlation (>90%) between the thickness and scattering length density of the SiO_*x*_ layer and to a lesser extent the roughness (which is of the same order of magnitude as the thickness for this layer), the thickness was fixed to 0.5 nm for temperatures >500 °C in order to stabilize the fits (fits of comparable quality could be achieved by conversely fixing the scattering length density instead). Owing to this high correlation and the fact that studying layers with a thickness below 1 nm is at the limits of XRR, the asymmetric error bar of the thickness is relatively large, with an upper error of the same order of magnitude as the thickness itself.

For *T* > 800 °C, the data could be equally well-described by a one-layer model taking only into account the poly-Si layer on top of the c-Si substrate. As the SiO_*x*_ layer significantly affects the reflectograms at lower temperatures (Figure S5), this indicates the disappearance of the SiO_*x*_ as a homogeneous, smooth layer along the poly-Si/c-Si interface with a substantially different electron density. We note that the quality of the fits for *T* > 800 °C decreases, which is both the case when using a one- and a two-layer model to describe the data. The lower quality of the fits, originating from an insensitivity of the minimization function to the small modulations on the data, is also reflected in the large error bars on the layer thickness and scattering length densities. Nevertheless, the trend of the densification of the poly-Si and SiO_*x*_ layer can also be concluded directly from the raw data: the fact that the fringes almost disappear for *T* > 800 °C implies that the electron density of these layer approaches the one of the c-Si substrate.

The last panel of Figure 5d presents a summation of the electrons in the sample excluding the substrate (integral of the SLD through the whole thickness) and is calculated from the fitted model and normalized to the as-deposited sample state. Different from panels b and c, this measure thus considers the total number of electrons in the sample and not its electron density. A decrease of this parameter in the range 300 °C < *T* < 500 °C would be associated with a loss of mass from the sample and could relate to, for example, hydrogen outgassing. This will be further discussed below in the context of the role of hydrogen in this thin-film stack.

**Figure 6:**
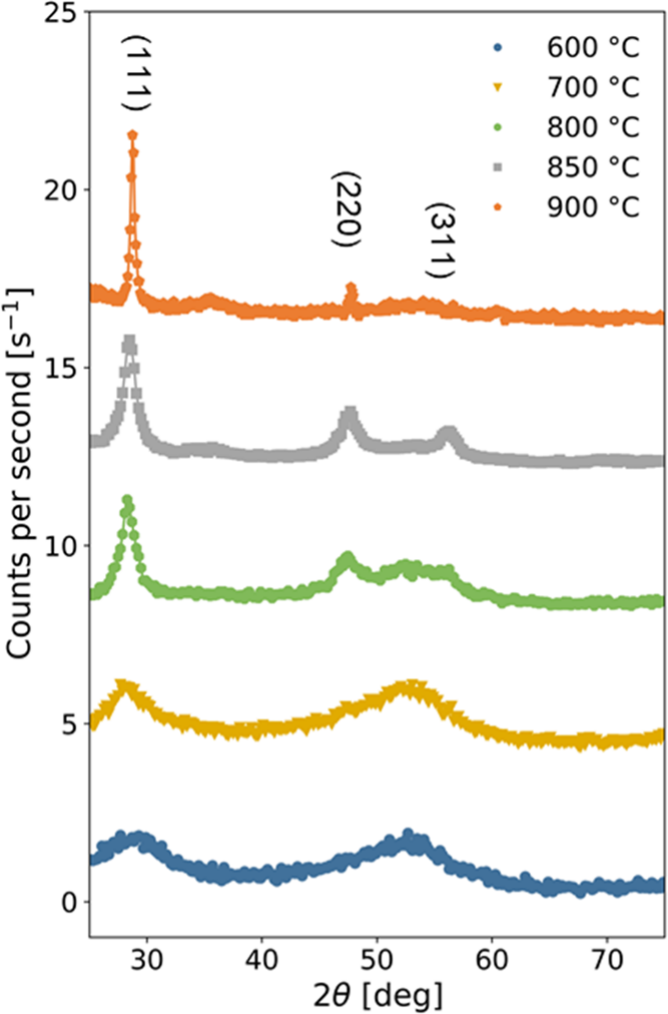
X-ray diffractograms measured during in situ annealing of an as-deposited sample from 600 to 900 °C. Diffractograms are vertically shifted for clarity.

GIXRD were also measured during in situ annealing of as-deposited samples between 600 °C and 900 °C, and the resulting diffractograms are illustrated in Figure 6. Diffractograms below 600 °C (not shown here) showed no significant changes compared to the one measured in the as-deposited state. We observe that the diffractogram measured at 600 °C and displayed in Figure 6 is still identical to the one measured on the as-deposited sample (Figure 3). Beginning at ∼800 °C, we observe the nucleation of crystalline domains through the appearance of three peaks at 2θ = 28.4, 47.3, and 56.1°; which can be attributed to the (111), (220), and (311) planes of the Si crystal structure. At 850 °C, the sample appears fully (poly-)crystalline with a flat background lacking any diffuse features. At 900 °C, the (111) and (220) peaks become significantly sharper and the relative intensity of the peaks changes, possibly indicating an increase in the crystallite size and a preferential grain-growth orientation, respectively.

## Discussion

In this part, we further discuss and propose some interpretations of the results obtained through the XRR and GIXRD analyses previously described.

### Structural Evolution of Poly-Si/SiO_*x*_ Contact during Annealing

**Figure 7:**
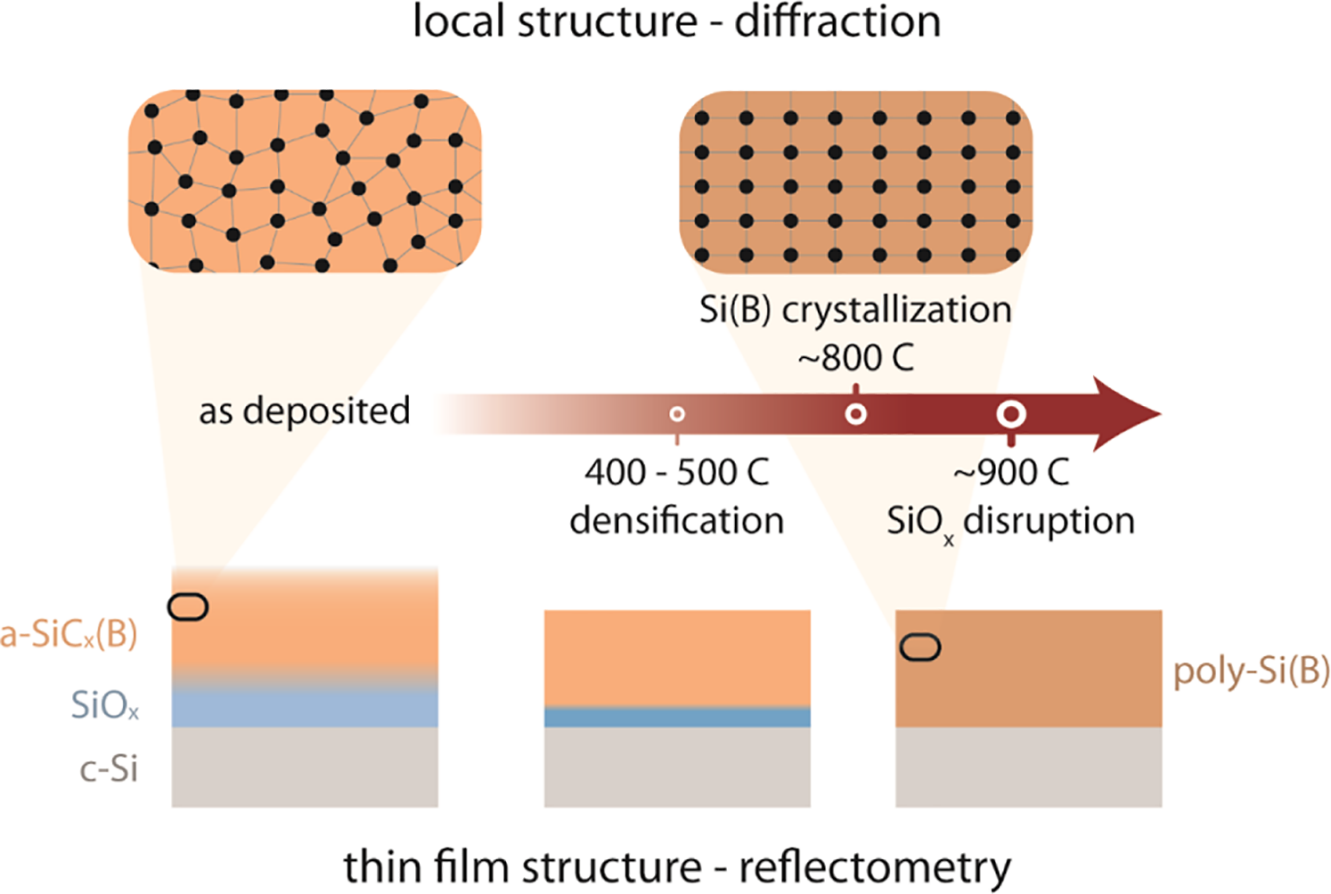
Schematic illustration of the structural evolution of the poly-Si/SiO_*x*_ contact as a function of temperature during annealing. We note that the figure is not to scale.

Figure 7 summarizes schematically the multiscale evolution of the multilayer structure as observed in our experiments. Through in situ XRR measurements, we observed that both poly-Si and SiO_*x*_ layers became thinner and denser during annealing up to 900 °C. For the poly-Si layer, this thinning and densification was most pronounced for 200 °C < *T* < 500 °C. The a-Si layers deposited by PECVD usually contain hydrogen due to the H-rich precursor gases used [usually SiH_4_, H_2_, and additional doping gas, e.g., B(CH_3_)_3_, PH_3_], which is known to out-diffuse during following annealing of the a-Si layer at high temperatures.^31−33^ The temperature range during which the out-diffusion of H occurs has been shown to depend on different parameters (e.g., doping of the a-Si layer).^31^ For B-doped a-Si layers similar to the ones studied here, the out-diffusion of H has been observed to occur between 200 and 450 °C,^31,32^ which is in good agreement with the temperature range during which we observed thinning and densification of the poly-Si layer, as well as overall loss of mass, by means of in situ XRR measurements (Figure 5d). Hydrogen out-diffusion has been linked to layer densification in previous studies.^34^ We note that the ∼5% loss of mass observed by XRR is likely too much to be only elemental hydrogen and would likely include contributions from precursor/inert gases trapped during PECVD. From *T* = 500 °C, we observed that the thickness and density of the poly-Si layer are then staying constant with the increase in temperature. Through in situ GIXRD, we observed the crystallization of the layer from a-Si to poly-Si starting from *T* = 800 °C. Overall these results indicate that there are two distinct mechanisms at play during high-temperature annealing of our poly-Si layer. First, for temperatures from 200 up to 500 °C, the layer becomes denser and thinner, most likely due to H out-gassing. Second, from *T* = 800 °C, the local structure of the layer starts reorganizing, leading to crystallization, which is however not associated to any significant changes of the layer’s macro structure (i.e., density and thickness).

For *T* > 500 °C, we observed a gradual decrease of the amplitude of the XRR fringes until almost complete vanishing for *T* > 800 °C. In this temperature range, the density of the poly-Si layer is practically equal to that of the c-Si substrate, so the amplitude of the fringes arises only from the density contrast between the SiO_*x*_ layer and the substrate. Thus, the loss of fringe amplitude for *T* > 800 °C indicates that the SiO_*x*_ layer is approaching the density of the poly-Si and c-Si substrate. Moreover, the SiO_*x*_ layer was observed to become continuously rougher with the increase in temperature during annealing (although we note that the roughness parameter is strongly correlated to the layer density in the fits of the data). These observations could be attributed to the disruption of the homogeneity of the thin SiO_*x*_ along the interface, leading to direct poly-Si/c-Si interfaces and potential epitaxial regrowth of the poly-Si layer from the c-Si substrate. This interpretation would be consistent with the overall loss of density contrast between the poly-Si/SiO_*x*_ stack and the c-Si substrate observed by XRR for *T* > 800 °C and also the sharpening of the XRD reflexes observed in Figure 6 for annealing at *T* = 900 °C.

### Structure–Property Relations

Several studies have already emphasized a disruption of the SiO_*x*_ layer at the interface after annealing at a high temperature in the range 800–1100 °C, for example, by means of TEM observations of the interface, changes of the transport barrier assessed by *T*-dependent IV measurements and based on a wet selective etching revealing poly-Si/c-Si direct interfaces (sometimes refer to as “pinholes”).^35−38^ The temperature at which the disruption of the SiO_*x*_ layer occurs depends among other things on the method used to grow the thin SiO_*x*_ layer.^36,39^ For chemically grown SiO_*x*_ layers similar to the ones used in this study, such a disruption of the SiO_*x*_ layer was already observed to occur after annealing at *T* ∼ 800–900 °C.^18,36,40^ More particularly, in ref (18), Figure 2 presents TEM analyses of samples processed in our lab, in a similar way than the ones of interest here. Through these TEM analyses, we observed that the disruption of the SiO_*x*_ interface occurred after long-annealing in between 800 °C and 900 °C, which is in good agreement with the temperature at which we observed XRR fringes vanishing in this study. Moreover, the disruption of the thin SiO_*x*_ at the interface has been observed to go along with a degradation of the surface passivation provided by the poly-Si contact, usually quantified by a loss of implied open-circuit voltage (iV_oc_) and an increase of the emitter recombination current density (*J*_0_).^36,41^ Here, we additionally evaluated the iV_oc_ and *J*_0_ after ex situ long annealing (before hydrogenation) in the range 800–900 °C by means of PCD on symmetrical samples. We observe an iV_oc_ maximum associated to a *J*_0_ minimum after annealing at 850 °C, which indicates that degradation of the surface passivation occurs between 850 °C and 900 °C for our sample’s structure. This result is consistent with the interpretation that vanishing of XRR fringes (Figure 4) is associated with the disruption of the SiO_*x*_ homogeneity at the interface. However, the still decent iV_oc_ value of 672 mV obtained after annealing at 900 °C indicates that the thin SiO_*x*_ layer may not be completely broken up along the interface, contrarily to what is suggested by in situ XRR experiments. This difference may come from an offset of ∼50 °C in the nominal temperature of the furnace used for long annealing compared to the XRD heating module used for in situ experiments, which is further discussed below. Moreover, the vanishing of fringes in reflectograms indicates that there is no more coherent layer that has a different electron density than the c-Si substrate, which may arises slightly before complete breakup of the SiO_*x*_ layer and thus complete loss of surface passivation.

After further hydrogenation through deposition of an H-rich silicon nitride (SiN_*y*_/H) layer and additional firing, iV_oc_ and *J*_0_ of 710 mV and 35 fA cm^–2^ were, respectively, obtained for samples annealed at 850 °C (Table 2), which roughly corresponds to state-of-the-art passivation properties for this structure.^32^ We note that for this type of symmetrical samples made from shiny-etched FZ c-Si wafers, we observed the formation of shallow electronic defect states in the bulk c-Si limiting the effective lifetime at first order under certain conditions (and thus leading to underestimation of the surface passivation properties provided by our poly-Si contact). For further details on this matter, the interested reader is referred to ref (42).

**Table 2:** iV_oc_ and *J*_0_ Measured by PCD on Samples Featuring Poly-Si/SiO_*x*_ Contacts on Both Sides After Long-Annealing at Various Temperatures

peak *T* (°C)	after annealing		after hydrogenation	
	iV_oc_ (mV)	*J*_0_ (fA cm^–2^)	iV_oc_ (mV)	*J*_0_ (fA cm^–2^)
800	624	125	682	22
850	676	100	710	35
900	646	275	672	73

Overall, our in situ experiments clearly demonstrate the sensitivity of the passivating contact to the peak temperature, especially in the practical range 700–900 °C. It seems that a high crystallinity of the poly-Si layer is a prerequisite to good passivation so that a delicate balance must be struck between a peak temperature high-enough to maximize crystallinity but low-enough to avoid the degradation of the SiO_*x*_.

### Effect of Different Thermal Profiles

Peak temperature is only one component of the thermal profile. Several recent studies reported the degradation of the surface passivation of the poly-Si contact after firing (i.e., high-temperature thermal process featuring fast heating and cooling rates), suggesting that the thermal profile to which the poly-Si/SiO_*x*_ contact is submitted strongly impacts its final structural and functional properties.^21,22^ In the following, we discuss our current understanding of the effects of the heating ramp rate, dwell time, and overall thermal budget by comparing samples annealed in different setups as described in detail in the experimental section (Table 1).

We note that while the results in terms of peak temperature are rather consistent between the firing and in situ annealing setups, the nominal peak temperatures of the long annealing setup seem to be overestimated by ∼50 °C (e.g., 850 °C in long annealing corresponds well to 800 °C in firing and in situ, see Figure S4 in the Supporting Information). This effect could be linked to the functional differences in the setups and/or a slight miscalibration of the temperature determination in one or more of the setups. Further investigation of this observation lies beyond the scope of this work but we suggest caution in interpreting the exact nominal temperatures we list here.

**Figure 8:**
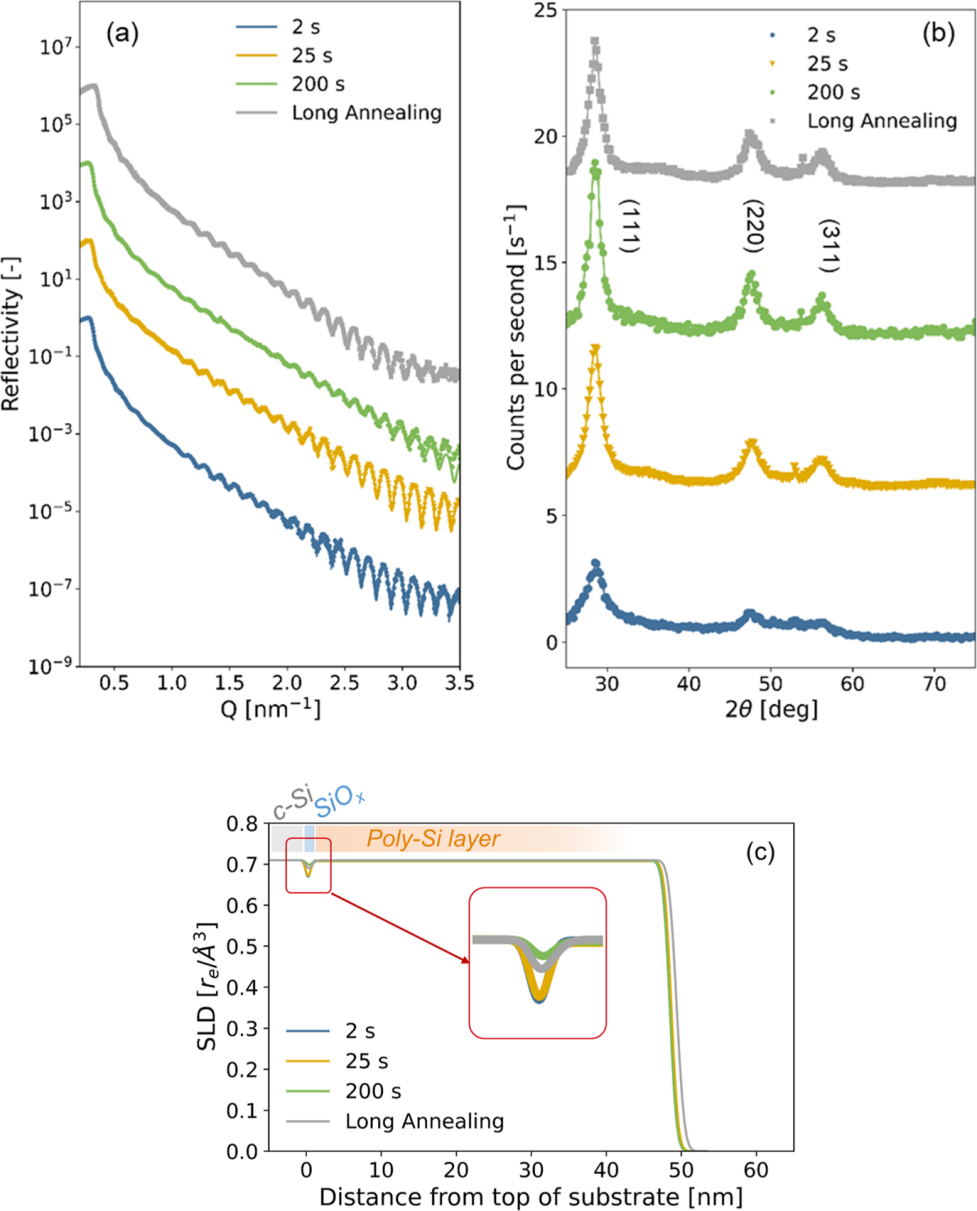
(a) X-ray reflectograms and (b) diffractograms of samples fired at 800 °C with different dwell times or long-annealed at 850 °C and (c) SLD profiles obtained from the fit of reflectograms plotted in (a). Structural parameters corresponding to the fits are tabulated in Table S3. Reflectograms in (a) and diffractograms in (b) are vertically shifted for clarity.

To investigate the impact of dwell time on the structure of the poly-Si contact, we performed additional ex situ XRR and GIXRD measurements after firing at 800 °C with various dwell times (2–200 s), which we compare to long annealing in Figure 8.

Comparing the XRR reflectograms in Figure 8a, we observe that fringes start visibly vanishing from a dwell time of 200 s, along with a lower density contrast between the thin SiO_*x*_ layer and the poly-Si layer and c-Si substrate in the associated SLD profiles displayed in Figure 8c. Moreover, the XRR results suggest that the poly-Si layer is about the same thickness and density after firing with different dwell times. Looking at the diffractograms obtained on the same samples in Figure 8b, we observe an increase of the poly-Si layer’s crystallinity after firing with the increase in dwell time. The firing results indicate that holding a longer dwell-time at 800 °C significantly increases the crystallinity of the poly-Si layer without impacting its thickness and density, which is in good agreement with in situ experiments presented above.

Finally, focusing on the SLD profiles at the SiO_*x*_ interface in Figure 8c, we observe similar profiles after firing with dwell times of 2 and 20 s but the longer dwell time of 200 s resulted in a significant loss of density contrast between the thin SiO_*x*_ layer and the poly-Si layer and c-Si substrate. This could indicate that the homogeneity of the thin SiO_*x*_ layer at the interface starts being compromised. In a recent study, we observed the degradation of the surface passivation of similar samples with the increase in firing dwell-time, with a more pronounced degradation for dwell-times superior to 30 s.^42^ This is in good agreement with the present XRR investigations and confirms that the vanishing of the fringes is linked to the breakup of the thin SiO_*x*_ along the interface.

Overall, our results suggest that increasing the dwell time at 800 °C (and so the overall effective thermal budget) has more impact on the integrity of the SiO_*x*_ layer at the interface of our samples than increasing the heating ramp rate. The detrimental impact of increasing the effective thermal budget of firing on the interface quality is in good agreement with observations made by Hollemann et al. in a study published recently.^21^ However, we note that in this study, they performed the firing step after previous activation of the poly-Si contact through a long-annealing step, whereas in the present study, the firing step was performed directly after deposition of the a-SiC_*x*_ layer.

Overall, the different results obtained in this part confirm once more the impact of the thermal profile on the final properties of both the poly-Si layer and the thin interfacial SiO_*x*_ layer, and thus, the importance of controlling such high-temperature processes when fabricating c-Si solar cells integrating poly-Si contacts.

## Conclusions

We have performed in situ XRR and GIXRD to monitor the structural changes of poly-Si/SiO_*x*_ contacts during high-temperature annealing. This allowed us to observe two distinct mechanisms during annealing of the poly-Si layer. First, for temperatures from 200 °C up to 500 °C, the layer becomes denser and thinner, most likely due to H out-gassing. Second, from *T* = 800 °C, the layer starts crystallizing, which is not associated to any significant changes of the layer’s macro structure (i.e., density and thickness). Moreover, we could monitor the disruption of the thin SiO_*x*_ layer in the range 850–900 °C, which is detrimental for the passivating properties of the poly-Si/SiO_*x*_ contact. Finally, results obtained on a broader range of thermal profiles, including firing, indicated that, for the poly-Si contact structure investigated here, a longer dwell-time is more detrimental to the structural integrity of the interfacial SiO_*x*_ layer than a faster heating ramp rate. Overall, our results emphasize once more the impact of high-temperature thermal budgets on the final properties of poly-Si contacts, and thus the importance of ensuring a good control of such high-temperature processes during the fabrication of c-Si solar cells integrating such contacts.

Moreover, this study demonstrates the robustness of combining different X-ray elastic scattering techniques (here XRR and GIXRD) to unravel the multiscale structural evolution of poly-Si contacts during high-temperature processes. These techniques present the unique advantage of being rapid, nondestructive, as well as applicable both in situ and on a large sample area. We believe these techniques are particularly adapted to the study of poly-Si contacts, and thus, in the near future, we will keep investigating their potential to reach a better understanding of the interrelation between poly-Si contacts’ final properties and their fabrication process. More particularly, follow-up studies will focus on the impact of the SiO_*x*_ layer properties (e.g., density, thickness) on the structural evolution of poly-Si contact during high-temperature annealing as well as on in situ hydrogenation with additional neutron reflectometry to directly correlate the structural changes to the H concentration.

## Supporting Information Available

Reflectograms of long-annealed (850 °C) samples measured ex situ with and without exposure to air, diffractograms of long-annealed samples at different incident angles, diffractograms of the bare c-Si substrate, fitted parameters obtained for all reflectograms presented in the main text, reflectograms and corresponding SLD of in situ and ex situ long-annealed samples, and simulated reflectograms for in situ annealed samples at different temperatures with and without thin SiO_*x*_ at the poly-Si/c-Si interface (PDF)

## Notes

The data underlying this study are openly available in the 4TU.ResearchData repository at doi.org/10.4121/19425749
