# Supplementary material for: In Situ Reflectometry and Diffraction Investigation of the Multiscale Structure of p-Type Polysilicon Passivating Contacts for c-Si Solar Cells
Source: ACS Appl Mater Interfaces. 2022 Mar 31;14(14):16413–23. doi: 10.1021/acsami.2c01225 (PMC9011350; doi:10.1021/acsami.2c01225)
Supplement: Supplementary file 1 — am2c01225_si_001.pdf [file am2c01225_si_001.pdf]

SUPPORTING INFORMATION

In situ reflectometry and diffraction investigation of the multiscale structure of p-type polysilicon passivating contacts for c-Si solar cells

Audrey Morisset [a], Theodosios Famprikis [b], Franz-Josef Haug [a], Andrea Ingenito [c], Christophe Ballif [a,c], Lars J. Bannenberg [b]**

[a]Ecole Polytechnique Fédérale de Lausanne (EPFL), Institute of Electrical and Microengineering (IEM), Photovoltaics and Thin Film Electronics Laboratory, Maladière 71b, 2002, Neuchâtel, Switzerland

[b]Department of Radiation Science and Technology, Faculty of Applied Sciences, Delft University of Technology, Mekelweg 15, 2629JB, Delft, the Netherlands

[c]CSEM PV-Center, Jacquet-Droz 1, 2002, Neuchâtel, Switzerland

Corresponding Authors

audrey.morisset@epfl.ch; l.j.bannenberg@tudelft.nl

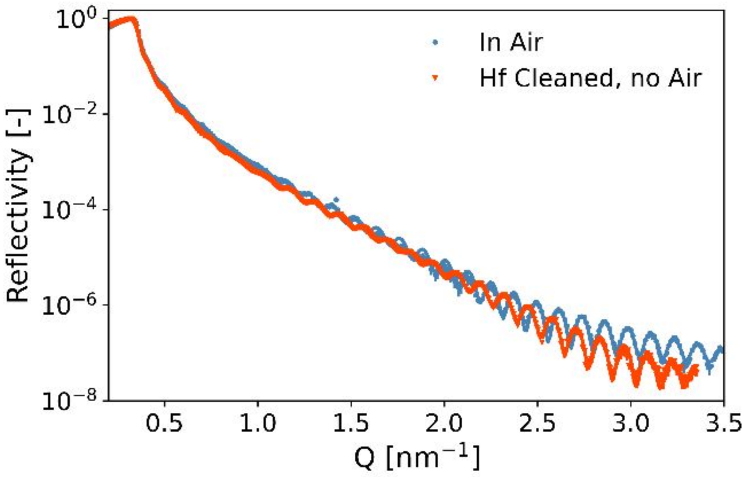

Figure S1: Reflectograms of long-annealed (850 ˚C) samples measured ex situ with and without exposure to air. The same poly-Si thickness is observed along with a ~2-3 nm native oxide on the sample measured in air.

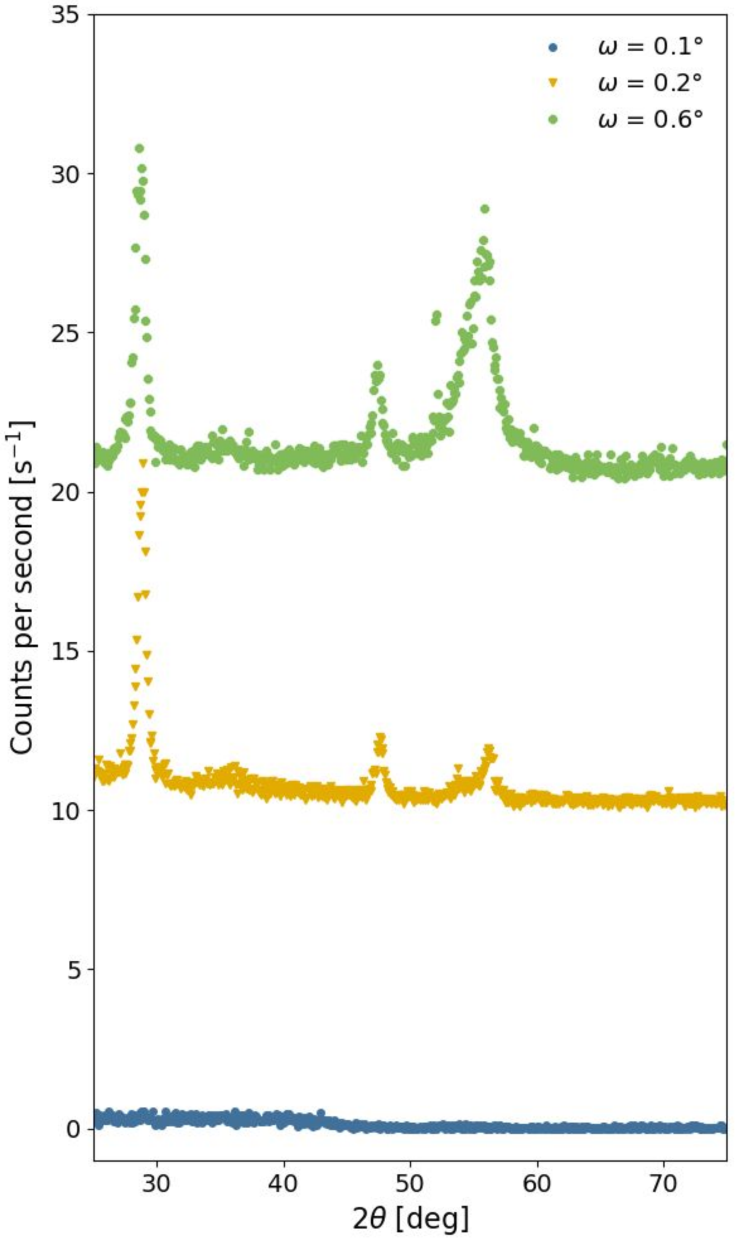

Figure S2: Optimization of grazing-incidence angle for GIXRD. Diffractograms of long-annealed sample (850 ˚C) at three difference incidence angles. Diffractograms are vertically shifted for clarity.

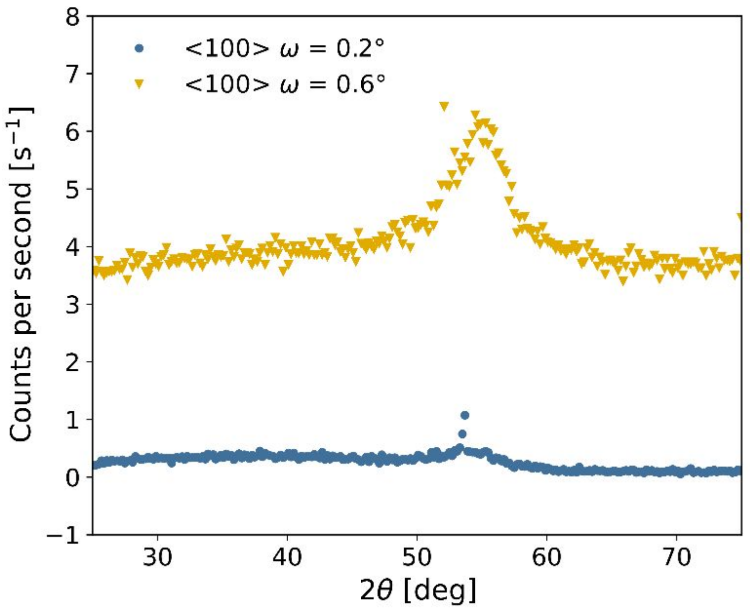

Figure S3: GIXRD measurements of bare <100>-oriented c-Si substrate at two different incidence angles to show that peak ~55 ° is from the c-Si substrate. Diffractograms are vertically shifted for clarity.

Table S1: Fitted parameters for the reflectograms shown in Figure 1 of the main text.

e

	Bare wafer	*As Deposited*	*ex situ Annealed*	*in situ Annealed*
poly-Si layer Thickness [nm]	-	58.0	48.8	49.0
poly-Si layer SLD [r_e/A^3]	-	0.629	0.708	0.699
poly-Si layer Roughness [nm]	-	1	0.8	0.8
SiO_x layer Thickness [nm]	-	0.5	0.6	0.5
SiO_x layer SLD [r_e/A^3]	-	0.358	0.676	0.667
SiO_x layer Roughness [nm]	-	0.3	0.3	0.3
c-Si Substrate SLD [r_e/A^3]	0.717	0.717	0.717	0.717
c-Si Substrate Roughness [nm]	0.3	0.3	0.3	0.3

Table S2: Fitted parameters for in situ reflectometry experiments shown in Figure 4 of the main text.

Temperature [°C]	50	200	300	350	400	450	500	550	600	700	800	850	900
poly-Si Thickness [nm]	57.5	57.5	56.7	55.7	54.2	52.7	50.5	49.5	49.1	49.0	49.0	49.1	48.5
poly-Si SLD [r_e/A^3]	0.632	0.627	0.635	0.638	0.645	0.664	0.668	0.691	0.695	0.691	0.699	0.699	0.706
poly-Si Roughness [nm]	0.9	1	0.9	0.9	0.8	0.8	0.6	0.7	0.7	0.7	0.8	0.7	0.7
SiO_x layer Thickness [nm]	0.6	0.5	0.5	0.5	0.5	0.5	0.5	0.5	0.5	0.5	0.5	-	-
SiO_x layer SLD [r_e/A^3]	0.318	0.318	0.315	0.318	0.467	0.473	0.534	0.522	0.597	0.634	0.667	-	-
SiO_x layer Roughness [nm]	0.3	0.3	0.3	0.3	0.3	0.3	0.5	0.5	0.3	0.3	0.3	-	-
c-Si Substrate SLD [r_e/A^3]	0.717	0.717	0.717	0.717	0.717	0.717	0.717	0.717	0.717	0.717	0.717	0.717	0.717
c-Si Substrate Roughness [nm]	0.3	0.3	0.3	0.3	0.3	0.3	0.3	0.3	0.3	0.3	0.3	0.3	0.3

Table S3: Fitted parameters for ex situ reflectometry experiments on fired samples shown in Figure 8 of the main text.

Method<br>Annealing Time [s]	Firing<br>2	Firing<br>25	Firing<br>200	Long annealing<br>n/a
poly-Si layer Thickness [nm]	48.0	48.2	47.8	48.8
poly-Si layer SLD [r_e/A^3]	0.716	0.714	0.716	0.708
poly-Si layer Roughness [nm]	0.8	0.7	0.7	0.8
SiO_x layer Thickness [nm]	0.5	0.5	0.5	0.6
SiO_x layer SLD [r_e/A^3]	0.643	0.646	0.694	0.676
SiO_x layer Roughness [nm]	0.3	0.3	0.3	0.3
c-Si Substrate SLD [r_e/A^3]	0.717	0.717	0.717	0.717
c-Si Substrate Roughness [nm]	0.3	0.3	0.3	0.3

Temperature [°C]	Ex situ<br>850	Ex situ<br>900	In situ<br>800	In situ<br>850
poly-Si layer Thickness [nm]	48.8	49.1	49.0	49.1
poly-Si layer SLD [r_e/A^3]	0.708	0.698	0.699	0.699
poly-Si layer Roughness [nm]	0.8	0.7	0.8	0.7
Interfacial layer Thickness [nm]	0.6		0.5	
Interfacial layer SLD [r_e/A^3]	0.676		0.667	
Interfacial layer Roughness [nm]	0.3		0.3	
Substrate SLD [r_e/A^3]	0.717	0.717	0.717	0.717
Substrate Roughness [nm]	0.3	0.3	0.3	0.3

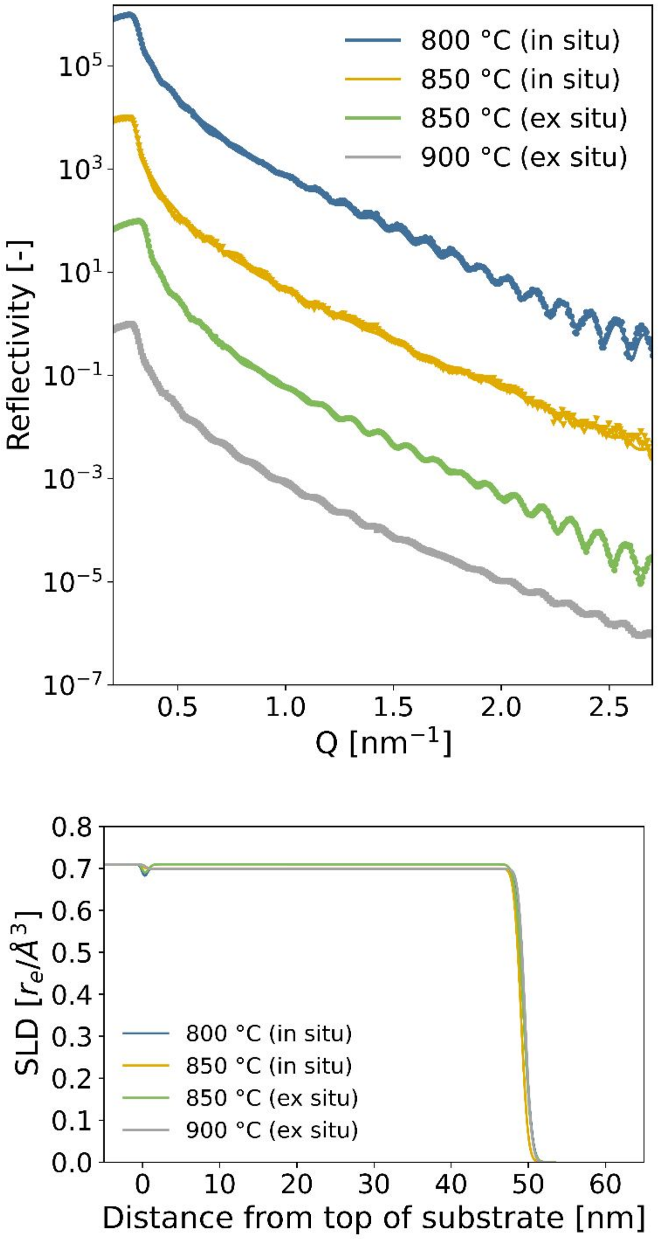

Figure S4: Reflectograms (top) and corresponding scattering length density profiles (bottom) of in-situ and ex-situ long-annealed samples demonstrating the ~50 ˚C offset in nominal temperature between setups. Reflectograms are vertically shifted by a constant factor (x100) for clarity.

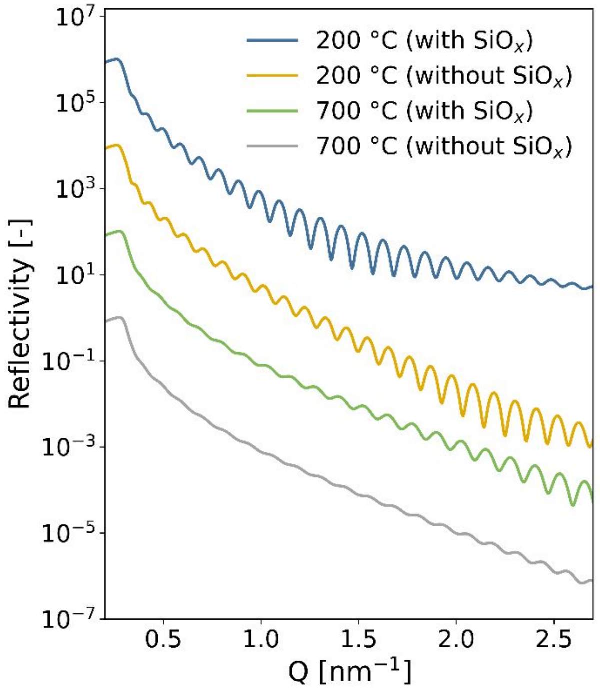

Figure S5: Simulations of in-situ annealed samples using the actual parameters obtained from the fits for T = 200 °C and 700 °C with and without a thin SiO_x layer at the interface to illustrate the sensitivity of XRR to the presence of the SiO_x layer.
